# Chelator-Free Copper-64-Incorporated Iron Oxide Nanoparticles for PET/MR Imaging: Improved Radiocopper Stability and Cell Viability

**DOI:** 10.3390/nano12162791

**Published:** 2022-08-14

**Authors:** Hye Min Jang, Myung Hwan Jung, Jae Sang Lee, Jun Sig Lee, In-Cheol Lim, Hyunsik Im, Sang Wook Kim, Sung-A Kang, Won-Je Cho, Jun Kue Park

**Affiliations:** 1 Korea Multi-Purpose Accelerator Complex, Korea Atomic Energy Research Institute, 181 Mirae-ro, Gyeongju 38180, Korea; 2 Division of Physics and Semiconductor Science, Dongguk University, Seoul 04620, Korea; 3 Korea Atomic Energy Research Institute, Daedeok-daero 989, Daejeon 34057, Korea; 4 Department of Advanced Materials Chemistry, Dongguk University, Gyeongju 38066, Korea; 5 Advanced Bio Convergence, Pohang Technopark, Pohang 37668, Korea

**Keywords:** superparamagnetic nanoparticles, magnetic relaxivity, PET-MRI, multimodal imaging, contrast agents, nanoparticles, silica core-shell

## Abstract

We have developed chelator-free copper-64-incorporated iron oxide (IO) nanoparticle (NPs) which have both magnetic and radioactive properties being applied to positron emission tomography (PET)-magnetic resonance imaging (MRI). We have found that the IO nanoparticles composed of radioactive isotope ^64^Cu may act as a contrast agent being a diagnostic tool for PET as well as a good *T*_2_ MRI nanoprobe due to their good *r*_2_/*r*_1_ ratio. Furthermore, we demonstrate that the ^64^Cu incorporation at the core of core-shell-structured IO NPs exhibits a good in vivo stability, giving us an insightful strategy for the design of a contrast agent for the PET-MRI system.

## 1. Introduction

To date, chelator-based radio-labeling is a widely used technique for the synthesis of various kinds of radio-labeled nanoparticles (NPs), which may be applied to positron emission tomography (PET) of a proven imaging technique. For example, porous silica NPs to attach fluorine-18 (^18^F, *t*_1/2_ = 109 min), copper-64 (^64^Cu, *t*_1/2_ = 12.7 h)-labeled carbon nanotubes, quantum dots, and superparamagnetic iron oxide NPs [1,2] have been developed for tumor-targeted cancer imaging and biodistribution studies. On the other hand, magnetic resonance imaging (MRI) as a noninvasive method gives good morphological or functional images for soft tissues. For diagnostic MRI procedures, however, we further need contrast agents (CAs) to enhance sensitivity and visuality for lesions [3,4]. For decades, a dual-modality probe for hybrid PET-MRI systems has been developed as an effective clinical diagnostic tool due to its synergetic combination of PET with high sensitivity and MRI with high resolution. It thus enables us to reduce the number of CAs injected into patients while fully benefitting both modalities [5].

To develop a stable radiopharmaceutical, it is important to select the best chelator with sufficient in vivo stability and to know the particular coordination chemistry. However, these are challenging tasks, so we need a simple but effective chelator-free strategy [4,6,7,8]. Recently, Chen et al. synthesized chelator-free zirconium-89-labeled mesoporous silica NPs enhancing their long-term in vivo integrity [9]. Wong et al. have also developed chelator-free copper-64-doped iron oxide (IO) NPs, but the dextran coating may easily be detached from the NPs when they are injected into the body, thus not perfectly avoiding the cytotoxicity effect [10].

## 2. Materials and Methods

### 2.1. Materials

To synthesize ^64^Cu-incorporated iron oxide (^64^Cu-IO) NPs, we purchased the reagents acetylacetone, sodium acetate, copper chloride, benzyl ether, iron acetylacetonate, lauric acid, dodecylamine, cyclohexane, ammonia hydroxide, and tetraethylorthosilicate from Alfa Aesar. In addition, reagents 1,2-hexadicanediol and igepal CO-520 were purchased from Sigma Aldrich (Burlington, MA, USA). ^64^Cu was supplied by Korea Institute of Radiological & Medical Sciences.

### 2.2. Production of Copper-64

The production of ^64^Cu was performed using concentrated nickel (^64^Ni). In order to make a nickel target for beam irradiation, a plating solution was prepared using ^64^Ni, and nickel sulfide (^64^NiSO_4_) was obtained from the concentrated ^64^Ni. The average energy of the proton beam was 12.3 MeV for the ^64^Ni(*p*,*n*)^64^Cu nuclear reaction, in which beam irradiation was performed using a 50 MeV accelerator (Scanditronix, Olvagen, Vislanda, Swden, 1985) at Korea institute of radiological&medical Sciences (KIRAMS). After beam irradiation, the target was separated from the target system; 10 mL of 6 N HCl solution was then taken in, stirred at 80 °C for 10 min to dissolve the concentrated material, and then cooled down to room temperature. This solution was adsorbed into a column with AG1-X8 (100–200 mesh) resin (4 cm × 10 cm), and, while flowing 10 mL of 6 N HCl solution into the column, collection of the eluted solution was in a prepared recovery container. After flowing the HCl solution, it was flowed into the column using 10 mL of high-purity distilled water for about 10 min, and the solution eluted in this process was collected in a sterile vial to recover ^64^Cu.

### 2.3. ^nat,64^Cu(acac)_2_ Synthesis

After mixing 5 mCi of ^64^Cu and 34 nmol of CuCl_2_, 2.5 equivalents of acetylacetone were added and then stirred for 5 min. Next, 3 equivalents of sodium acetate were added to crystallize, and the mixture was then stirred at 80 °C for 15 min, followed by cooling at room temperature. After centrifugation to separate the solvent and the solute, it was washed three times with benzyl ether. Finally, ^64^Cu recovery was measured by a dose calibrator.

### 2.4. Core-Shell Structures of IO and ^64^Cu-IO

The ^64^Cu acetylacetonate (^64^Cu(acac)_2_) total, 0.98 g iron acetylacetate, 0.387 g 1,2-hexadecanediol, 0.3 g lauric acid, 0.344 mL dodecylamine, and 100 mL benzyl ether were stirred at 180 °C for 2 h under argon gas condition. After the reaction, the resulting ^64^Cu-IO NPs were collected after centrifugation and washing and were then dried in an oven. A total of 10 mL of igepal CO-520 was dispersed in 110 mL of cyclohexane and sonicated for 10 min. Then, 5 mL of ^64^Cu-IO solution (2.5 mg/mL cyclohexane) was added to the above solution with continuous stirring, followed by ultrasonication for 1 h. Subsequently, 2 mL of ammonium hydroxide was added to the above mixture solution. Finally, 1.4 mL of tetraethylorthosilicate (TEOS) was added. After stirring for 2 h, the resulting ^64^Cu-IO core-shell NPs at SiO_2_ were collected after centrifuging and washing, and then they were dried in the oven.

### 2.5. Characterization

The size and shape of ^64^Cu-IO NPs were examined using scanning electron microscopy (SEM) (JEOL, Tokyo, Japan) and transmission electron microscopy (TEM) (HITACHI, Tokyo, Japan). For the elemental analysis, an element composition mapping was performed using an energy dispersive X-ray attachment (EDS) equipped with SEM. Inductive coupled plasma mass spectroscopy (ICP-MS) (Perkin Elmer, Waltham, MA, USA) was carried out to measure the element concentration of ^64^Cu-IO. The distributions of the particle sizes of IO and ^64^Cu-IO NPs were examined by a dynamic light scattering (DLS) analyzer (Nanotrac wave, Montgomeryville and York, PA, USA). The Fourier transform infrared spectroscopy (FTIR) (Perkin Elmer, Waltham, MA, USA) was performed to confirm the functional groups on the surface of the NPs. The structure of ^64^Cu-IO was examined by X-ray diffraction (XRD) in a range of 5–90° as a function of the diffraction angle 2θ by using a MiniFlex 600 (RIGAKU, Tokyo, Japan) diffractometer with 3 kW monochromatic Co radiation (λ = 1.79 Å). To investigate the chemical state of the NPs, X-ray photoelectron spectroscopy (XPS) was employed. The XPS spectra were measured by a PHI 5000 VersaProbe spectrometer (Ulvac-PHI, Osaka, Japan) equipped with a monochromatized Al Kα (1486.6 eV) X-ray source. All of the binding energies were calibrated with the C 1s peak at 284.6 eV.

### 2.6. Magnetism and Relaxivity

The magnetization curves were obtained from a superconducting quantum interference device (SQUID) magnetometer of Quantum Design MPMS at Korea Basic Science Institute (KBSI). M(T) curves were recorded from 2 K to 300 K under 500 Oe applied field and under zero-field. M(H) curves were made in the field range of ± 70 kOe at temperatures of 5 K and 300 K. The relaxivity measurements were made using a 4.7 T and a 9.4 T MRI instrument systems. MRI experiments were performed on a 4.7 T animal MRI scanner (BioSpec 47/40; Bruker, Karlsruche, Germany) and a 9.4 T animal MRI scanner (Agilent, Santa Clara, CA, USA) with a 40 mm volume coil at Korea Basic Science Institute in Ochang. *T*_1_ relaxivity was obtained using the spin echo pulse sequence with a variable repeat time, and *T*_2_ relaxivity was obtained using the Carr–Purcell–Meiboom–Gill sequence at 4.7 T and 9.4 T.

### 2.7. Cytotoxicity Effects

The cytotoxicity (in vitro test) was performed using human umbilical-vein-endothelial cells (HUVECs). The HUVECs were purchased from Promocell company (Sickingenstrabe, Heidelberg, Germany) and cultivated in an incubator under 37 °C and 5% CO_2_ using EGM-2 as the culture medium. To estimate the cell viability, we used the cell counting kit-8 (CCK-8) (Dojindo Company, Gumamoto, Japan). The CCK-8 is based on the absorbance analysis that forms an orange-colored formazan through a dehydrogenase in the cell. We cultivated the cells under 37 °C and 5% CO_2_ for 24 h in an incubator to attach them to the bottom of the plate, each seeding as many as 2 × 10^3^ cells per well in a 96-well plate. Then, we introduced the NPs onto HUVECs, followed by the CCK-8 treatments after 6 h, 12 h, and 24 h. Lastly, 2 h after the treatments, we performed the centrifuge of the culture medium and then removed the NPs that may affect the absorbance, followed by placing them in a new 96-well plate to measure the survival rate at a wavelength of 450 nm with a microplate reader (Biotek, Winooski, VT, USA). The cell-survival rate was given by the ratio of absorbance of the test group to that of the control group.

### 2.8. Simulated Body Fluid (SBF) Tests

SBF is a solution with ion concentration, composition, and pH values similar to those of blood plasma. To ensure an in vivo stability of SiO_2_ formed on the NPs, we immersed the NPs into 10 mg/mL SBF for 1 h, 2 h, 1 d, 3 d, 5 d, and 7 d. The samples, depending on the immersing time, were then examined by TEM.

### 2.9. PET Experiments

Male *BALB/c* mice aged 11 weeks were acclimatized to a laboratory environment for 2 weeks. The mice were housed under standard conditions of light, temperature, and humidity. Human lung adenocarcinoma cells of A549 were used for injection in the mice. Mice thus were infected with A549 as many as 5 × 10^5^ cells by intraperitoneal injection. We grew the *BALB/c* male mice with cancer cells for two weeks. We then expanded the tail by immersing it in warm water. The 10 μL NPs dispersed in saline were then injected into the tail-vein using an insulin injector, and then we waited for 1 h for them to circulate into the body. After injection, we placed *BALB/c* in the chamber and performed primary anesthesia at 1% concentration. Then we connected the small animal’s anesthesia cone to the *BALB/c* respiratory system, so that the isoflurane administration to the *BALB/c* respiratory system was maintained at a 3% concentration during measurement. After that, we measured the PET image with the imaging chamber in which anesthetic drugs were injected into the respiratory organs of the mice. The radioactivity of ^64^Cu using PET/SPECT/CT (Siemens Medical Solutions USA, Inc., Malvern, PA, USA) was obtained after 1 h. PET/SPECT/CT images were analyzed by using Invent Acquisition workplace software (syngo.via for MI, Ver. 1.5; Siemens Preclinical Solutions, Munich, Germany). All experimental procedures were approved by the Institutional Animal Care and Use Committee (IACUC) of Pohang University of Science and Technology (POSTECH). All surgery was performed under the isoflurane anesthesia system, and all efforts were made to minimize suffering.

## 3. Results and Discussion

### 3.1. Synthesis and Characterization of ^64^Cu-IO@SiO_2_ NPs

**Figure 1:**
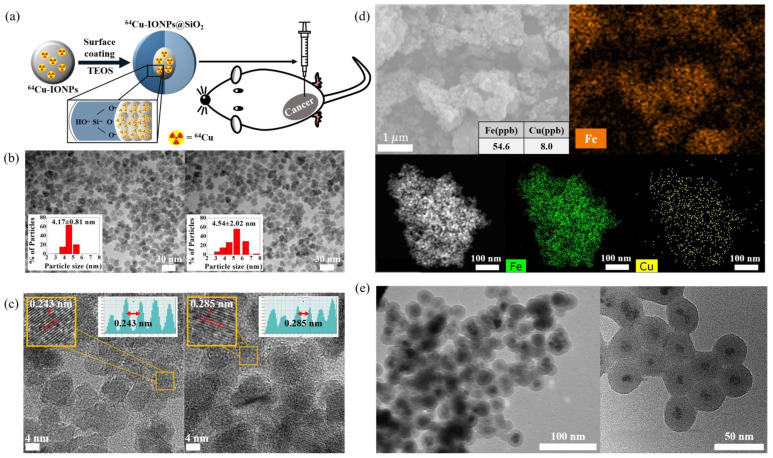
(**a**) Schematic cartoon for the experimental process for synthesizing the NPs, coating with TEOS, injecting the NPs into *BALB/c* mice, and PET and MRI imaging. (**b**) TEM images of IO and ^64^Cu-IO NPs. The inset displays the size distribution acquired by measuring at least 200 NPs from the corresponding TEM image of each sample. (**c**) The lattice constants of IO and ^64^Cu-IO NPs obtained from TEM images for both samples. (**d**) SEM images of IO NPs (upper panel) and TEM images of chelator-free ^64^Cu-IO NPs (lower panel) with elemental mapping analysis confirming the presence of Fe and Cu ions. (**e**) TEM images for core-shell-structured IO (left) and ^64^Cu-IO NPs (right).

Figure 1a displays a schematic cartoon that presents the structure of the NPs and the process of injecting them into a *BALB/c* mouse. Figure 1b shows TEM and SEM images of the as-synthesized IO and ^64^Cu-IO NPs. The mean sizes of the particles were to be 4.17 ± 0.81 nm and 4.54 ± 2.02 nm for IO and ^64^Cu-IO NPs, respectively, as observed by TEM images, where they dispersed well without any aggregation [9,10,11]. In Figure 1c, the lattice constant was estimated to be 0.243 nm and 0.285 nm for the IO and ^64^Cu-IO NPs, respectively, due to a slightly larger radius of Cu compared to Fe, implying that Cu was well incorporated into IO. EDS mapping analysis confirmed that Fe and Cu presented well in the individual ^64^Cu-IO NPs (Figure 1d). The Cu concentration of the ^64^Cu-IO NPs was assessed with an ICP-MS, confirming that the ratio of Fe to Cu is about 7 to 1. We note that, in our ^64^Cu-IO NPs, the radioisotope ^64^Cu and the stable ^nat^Cu were co-incorporated into the IO NPs. Figure 1e exhibits the IO and ^64^Cu-IO NPs surrounded by the shell consisting of TEOS. As shown in Figure 1e, the core NPs are well surrounded by a water-soluble shell structure of SiO_2_, supporting our NPs to become a proper contrast agent for the living cells [2,12,13,14]. To avoid metallic toxicity for the mouse models, the shell structure has been developed to be adsorbed on the surface of the core NPs, giving us IO@SiO_2_ and ^64^Cu-IO@SiO_2_ NPs [15,16].

**Figure 2:**
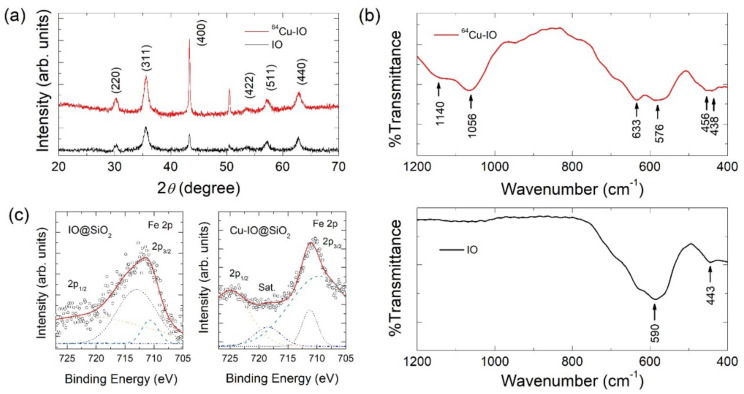
(**a**) XRD patterns and (**b**) FTIR spectra for chelator-free ^64^Cu-IO (upper panel) and IO NPs (lower panel). (**c**) XPS spectra of IO@SiO_2_ (left) and Cu-IO@SiO_2_ (right) for Fe 2p.

Figure 2a shows the XRD patterns of IO and ^64^Cu-IO NPs, confirming that the observed diffraction peaks correspond to the cubic spinel structure (ICSD card no. 98-015-8743) [2,15]. No obvious changes in the diffraction peaks were found upon the doping of both ^64^Cu and ^nat^Cu into the IO NPs. In Figure 2b, FTIR spectra for the IO and ^64^Cu-IO are displayed. From a peak at 443 cm^−1^, we found that a small amount of Fe_2_O_3_ together with Fe_3_O_4_ was synthesized. The introduced Cu ions in ^64^Cu-IO NPs may give rise to a small shift of the peak from 443 cm^−1^ to 456 cm^−1^ [17]. The Cu–O vibrations were also shown from the peaks of 633 cm^−1^, 1056 cm^−1^, and 1140 cm^−1^ for the ^64^Cu-IO sample [18]. In XRD, however, the peaks of Fe_2_O_3_ and Fe_3_O_4_ would be superposed, because they have the same crystal structure. Thus, we could not find the introduced Fe_2_O_3_ phase in XRD data [7,19].

In Figure 2c, we display core-level XPS spectra of Fe 2p for IO@SiO_2_ and Cu-IO@SiO_2_ NPs, ranging from 727 eV to 705 eV. The spectra were well fitted with four Gaussian lines after the Shirley background was subtracted. For both samples, two main peaks corresponding to Fe 2p_1/2_ and Fe 2p_3/2_ appeared at ~725 eV and ~711 eV, respectively. In comparison of these two spectra, however, the data of Cu-IO@SiO_2_ NPs exhibit more narrowed linewidth than that of IO@SiO_2_ NPs, also giving us a greater satellite peak of Fe 2p_3/2_ located at 718.5 eV, indicative of the presence of Fe^3+^. Two separated peaks at 709.5 eV and 711.2 eV in Fe 2p_3/2_ may be attributed to Fe^3+^ from Fe_3_O_4_ phase and Fe_2_O_3_ phase [20,21] as in FTIR spectra. Cu 2p, O 1s, and survey spectra can be found in Figure S1 in the Supplementary Materials.

### 3.2. Magnetic Properties of NPs

**Figure 3:**
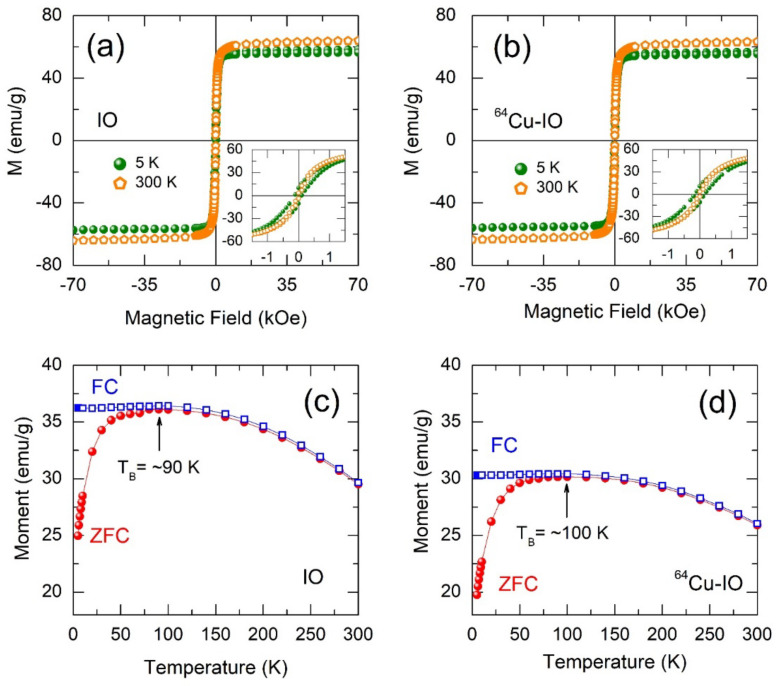
Mass magnetization of the (**a**) IO and (**b**) chelator-free ^64^Cu-IO NPs obtained using a SQUID magnetometer. The insets of (**a**,**b**) show the magnified curves exhibiting a perfect superparamagnetic behavior at 300 K for both samples. The remanent magnetization and coercive field at 5 K were 10.3 emu/g and 100 Oe for (**c**) IO, and 10.8 emu/g and 200 Oe for (**d**) chelator-free ^64^Cu-IO, respectively. Zero-field-cooled (ZFC) and field-cooled (FC) temperature-dependent magnetization curves (M-T) for IO and chelator-free ^64^Cu-IO NPs under an applied magnetic field of 500 Oe. *T*_B_ represents the blocking temperature.

In Figure 3a,b, the superparamagnetic behavior is observed for the IO and ^64^Cu-IO NPs at 300 K, whereas ferromagnetic behavior is obvious at 5 K for both samples with a remanent magnetization and a coercive field (insets of Figure 3a,b). It should be noted that, in both samples, the magnetization at 300 K is greater than that at 5 K, which may be due to the spins from the canted anti-ferromagnet (or weak ferromagnet) at 5 K resulting in spin-glass-like behavior [22]. To confirm the behavior in these samples, we performed the zero field-cooled (ZFC) and field-cooled (FC) measurements. Figure 3c,d exhibit M-T curves at ZFC and FC conditions of IO and ^64^Cu-IO NPs. A similar behavior between ZFC and FC magnetization was observed for both samples, in which the blocking temperature (*T*_B_) in the ZFC curve was comparably observed at ~90 K and ~100 K for IO and ^64^Cu-IO NPs, respectively. The *T*_B_ can be defined as the temperature above which thermal energy overcomes anisotropy energy, thereby permitting the particles to rotate to their thermal equilibrium direction [23,24]. The splitting between the ZFC and FC curves denotes a typical behavior of the superparamagnetic system.

A slight increase in *T*_B_ of ^64^Cu-IO may be due to slightly larger sizes of NPs, since *T*_B_ is proportional to the particle volume *V* according to the relation, [24] *T*_B_ = *KV*/ln(*τ*_M_/*τ*_0_)*k*_B_, where *K* is the magnetic anisotropy constant, *τ*_M_ is the measuring time window of the experimental method, and *τ*_0_ is the attempt time. In the FC curves for both samples, very weak variation below *T*_B_ indicates the presence of spin-glass-like nature due to dipole-dipole interactions between the NPs [23,24].

### 3.3. MR Relaxivities of IO and ^64^Cu-IO NPs at 4.7 T and 9.4 T

**Figure 4:**
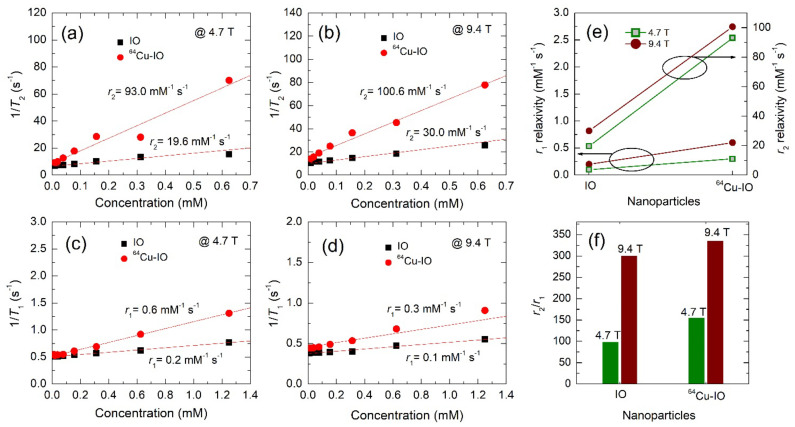
Transverse (*r*_2_) relaxivity of IO and chelator-free ^64^Cu-IO NPs at (**a**) 4.7 T and (**b**) 9.4 T. Longitudinal (*r*_1_) relaxivity of IO and ^64^Cu-IO NPs at (**c**) 4.7 T and (**d**) 9.4 T. (**e**) The *r*_1_ and *r*_2_ relaxivities at 4.7 T and 9.4 T for IO and ^64^Cu-IO. (**f**) The *r*_2_/*r*_1_ ratios for IO and ^64^Cu-IO are compared at 4.7 T and 9.4 T.

The *r*_2_ (=1/*T*_2_) and the *r*_1_ (=1/*T*_1_) relaxivities of IO and ^64^Cu-IO NPs were investigated at 4.7 T and 9.4 T, as shown in Figure 4. In Figure 4a–d, all the *r*_2_ and the *r*_1_ relaxivities were well fitted to a linear function with good reproducibility. In lower and higher fields, both *r*_2_ and *r*_1_ relaxivities of ^64^Cu-IO were greater than those of IO. Comparing the *r*_2_/*r*_1_ ratios between IO and ^64^Cu-IO in Figure 4f, the latter can thus be a better *T*_2_ dominant CA. We now may consider how *r*_2_ and *r*_1_ relaxivities of ^64^Cu-IO are greater than those of IO. In principle, *r*_2_ relaxivity may depend on the two correlation times: the diffusion correlation time, *τ*_D_ = *r*_c_^2^/*D*, and the static correlation time, 1/∆*ω*, where *r*_c_ is the radius of the NPs, *D* is the diffusion coefficient of water, and ∆*ω* is the difference in Larmor frequency of the particle and that at infinity [25,26]. Considering our smaller particle size of ~4 nm, *r*_2_ relaxivity may be in a regime where motional narrowing is satisfied, which is proportional to *τ*_D_. In this regime (*τ*_D_ ≪ 1/∆*ω*), diffusion is faster than a spatial variation of local field inhomogeneity induced by a single particle. For *r*_1_ relaxivity, the relaxation enhancement of ^64^Cu-IO compared to IO may be due to a higher diffusion of water protons along with the larger superparamagnetic particles. A greater *r*_2_ value in ^64^Cu-IO compared with IO may thus be associated with a slightly larger mean size of the particles (see Figure 1a). Consequently, Cu ions introduced in IO NPs may lead to slightly better performance as a *T*_2_ CA.

### 3.4. Biocompatibility of Cu-IO@SiO_2_ In Vitro

**Figure 5:**
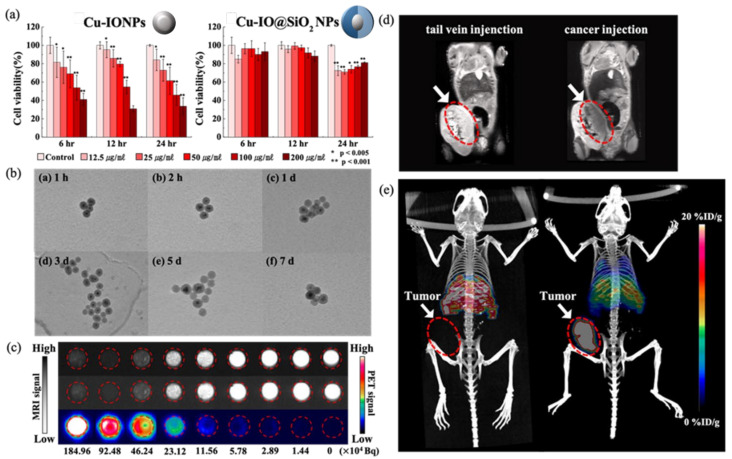
(**a**) Cell viability results examined by the CCK-8 using HUVECs as a function of time with varying concentrations to assess the cytotoxicity. (**b**) Representative TEM images for Cu–IO@SiO_2_ NPs incubated in SBF at 37 °C for (b-a) 1 h, (b-b) 2 h, (b-c) 1 d, (b-d) 3 d, (b-e) 5 d, and (b-f) 7 d. SBF results confirm the stability of core-shell structures when the NPs are injected into the body. (**c**) MRI and PET images were measured with varying concentrations of NPs which were diluted half consecutively starting from 184.96 × 10^4^ *Bq* down to 1.44 × 10^4^ *Bq* to examine the contrast performance. (**d**) MR images and (**e**) PET images were measured in 3 h after injecting the NPs into the *BALB/c* mice. Intravenous injection at the tail (left of (**d**,**e**)) and direct injection into cancer (intratumoral) (right of (**d**,**e**)) of each mouse model. Tumor: white arrows; tumor size and position: red dotted ovals.

An essential precondition for biomedical applications is to obtain NPs with a hydrophilic surface property. We examined the cytotoxicity of the HUVECs on IO and Cu-IO NPs by CCK-8 assay with various concentrations at 6 h, 12 h, and 24 h post-injection. In Figure 5a, we display the cell viabilities of the Cu-IO NPs with and without the shell structure of SiO_2_. In the case of NPs without the hydrophilic shell structure, as expected, endothelial cell viabilities gradually decrease with the increasing concentration of the NPs. The asterisks indicated a significant difference relative to the control (*, *p* < 0.005; **, *p* < 0.001) based on the Student’s *t*-test. Error bars represent standard deviation (*n* = 3). The cell viabilities exhibit a similar trend depending on the delaying time, but they exhibit the lowest viability at 24 h post-injection. On the other hand, the NPs with the shell structure, Cu-IO@SiO_2_, exhibit no appreciable change in the cell viabilities with varying concentrations of the NPs and the delaying time. Figure 5b shows representative TEM images for Cu-IO@SiO_2_ NPs which are incubated in SBF at 37 °C for 1 h, 2 h, 1 d, 3 d, 5 d, and 7 d. Regardless of the incubating time, we cannot find any destruction in the shape of the surface and any aggregation for the NPs. Based on these two distinct experiments, we may ensure that our Cu-IO@SiO_2_ NPs have high biocompatibility and long-term stability, making them suitable for biomedical applications.

### 3.5. PET/MR Images of ^64^Cu-IO@SiO_2_ NPs

PET and MRI data of ^64^Cu-incorporated IO@SiO_2_ NPs are displayed in Figure 5c. Obviously, the PET signal increases with increasing ^64^Cu ions up to 50 μCi/mL, together with increasing concentration of ^64^Cu-IO@SiO_2_ NPs. As shown in Figure 5c, ^64^Cu-IO@SiO_2_ NPs excellently work for the MRI and PET images without any possible interference when combined into a single nanoparticle [27,28,29]. In Figure 5d, we display MR images of *BALB/c* mice when NPs were intravenously injected at the tail and directly injected into the cancer of the mice. When NPs were intravenously injected at the tail, the image is bright around the tumor as denoted by a red circle due to the absence of the NPs (left image). By direct injection, in contrast, the image around the tumor becomes dark when the NPs are around it, as shown in the right image of Figure 5d. However, we may not discriminate differences between these two images except for the tumor, thus not ensuring where other NPs are. Here, we predict that PET images over MR images may further provide more clear anatomical information, when using a dual-modality probe. To ensure whether PET provides clear anatomical information, we also performed the PET measurement. For the distribution of ^64^Cu-IO@SiO_2_ NPs into a body, we injected the NPs into the tail of healthy *BALB/c* mice living 11 weeks after they were born (left image in Figure 5e).

The NPs go towards the heart and then circulate to the whole body of the mouse. Within 3 h, we performed the PET imaging, and then successfully confirmed that the NPs are in the liver and the lung after they circulate in the body of a mouse (left image). Note that in this work we do not allow the ability of surface functionalization with specific targeting of biomarkers for tumors. In addition, we examined the PET image by injecting the NPs directly into the tumor in a mouse. As shown in the right image of Figure 5e, we successfully observed that the NPs spread around the liver and the lung after the circulation for 3 h, demonstrating that they become a good CA for PET (see Figure S2 in the Supplementary Materials). We note that a key factor in the assessment for pharmacokinetics is fast circulation within 3 h after injection [30,31,32]. The CAs staying mainly at the tumor despite their circulation in the body may be due to an effect of enhanced permeability retention [33,34]. Upon an increasing monitoring time, the radioactivity may go towards the liver and the spleen, and then would be cleared into the urine via the kidney [35,36]. We thus clearly observed a discernible image upon a distinct injection into the mice, obtaining obvious anatomical information.

## 4. Conclusions

In summary, we have developed chelator-free copper-64-incorporated iron oxide NPs with the core-shell structure for PET-MR imaging. We successfully synthesized ^64^Cu-IO NPs surrounded by the shell consisting of TEOS, exhibiting good biocompatibility and stability, as proved by in vitro SBF test and PET imaging. In both IO and ^64^Cu-IO NPs, superparamagnetic behaviors were equivalently observed, giving comparable blocking temperatures at ~90 K and ~100 K for the IO and ^64^Cu-IO NPs, respectively. In MR relaxivity, it is revealed that Cu ions introduced in IO NPS may lead to slightly better performance as a *T*_2_ CA, due solely to their slightly larger mean size of the particles. In MR imaging, we compared the images of *BALB/c* mice between intravenous injection at the tail and direct injection into cancer, in which intravenous injection may further need PET images to acquire clear anatomical information. From PET images, we complementarily observed that our NPs spared around the liver and the lung by intravenous injection, demonstrating that they become a good dual-modality probe for hybrid PET-MRI systems.

## Data Availability

All data presented in this study are available upon request from the corresponding author.

## Supplementary Materials

The following supporting information can be downloaded at: https://www.mdpi.com/article/10.3390/nano12162791/s1, Figure S1: Survey data of (a) IO@SiO_2_ and (b) Cu-IO@SiO_2_. Fe 2p spectra of (c) IO@SiO_2_ and (d) Cu-IO@SiO_2_. Cu 2p spectra of (e) IO@SiO_2_ and (f) Cu-IO@SiO_2_. O 1s spectra of (g) IO@SiO_2_ and (h) Cu-IO@SiO_2_.; Figure S2: PET images measured immediately and in 3 h after intratumoral injection (direct injection into cancer) of the NPs for the *BALB/c* mice.
