# Supplementary material for: Chelator-Free Copper-64-Incorporated Iron Oxide Nanoparticles for PET/MR Imaging: Improved Radiocopper Stability and Cell Viability
Source: Nanomaterials (Basel). 2022 Aug 14;12(16):2791. doi: 10.3390/nano12162791 (PMC9416411; doi:10.3390/nano12162791)
Supplement: Supplementary file 1 [file nanomaterials-12-02791-s001.zip › nanomaterials-1856954-supplementary.pdf]

Supplementary Materials

Chelator-Free Copper-64-Incorporated Iron Oxide Nanoparticles for PET/MR Imaging: Improved Radiocopper Stability and Cell Viability

Hye Min Jang [1,2], Myung Hwan Jung [1], Jae Sang Lee [1], Jun Sig Lee [3], In-Cheol Lim [3], Hyunsik Im [2], Sang Wook Kim [4], Sung-A Kang [5], Won-Je Cho [1,*] and Jun Kue Park [1,*]

1 Korea Multi-Purpose Accelerator Complex, Korea Atomic Energy Research Institute, 181 Mirae-ro, Gyeongju 38180, Korea
2 Division of Physics and Semiconductor Science, Dongguk University, Seoul 04620, Korea
3 Korea Atomic Energy Research Institute, Daedeok-daero 989, Daejeon 34057, Korea
4 Department of Advanced Materials Chemistry, Dongguk University, Gyeongju 38066, Korea
5 Advanced Bio Convergence, Pohang Technopark, Pohang 37668, Korea
* Correspondence: wonje59@kaeri.re.kr (W.-J.C.); jkuepark@kaeri.re.kr (J.K.P.)

X-ray photoelectron spectroscopy (XPS) results of IO@SiO_2 and Cu-IO@SiO_2 nanoparticles (NPs)

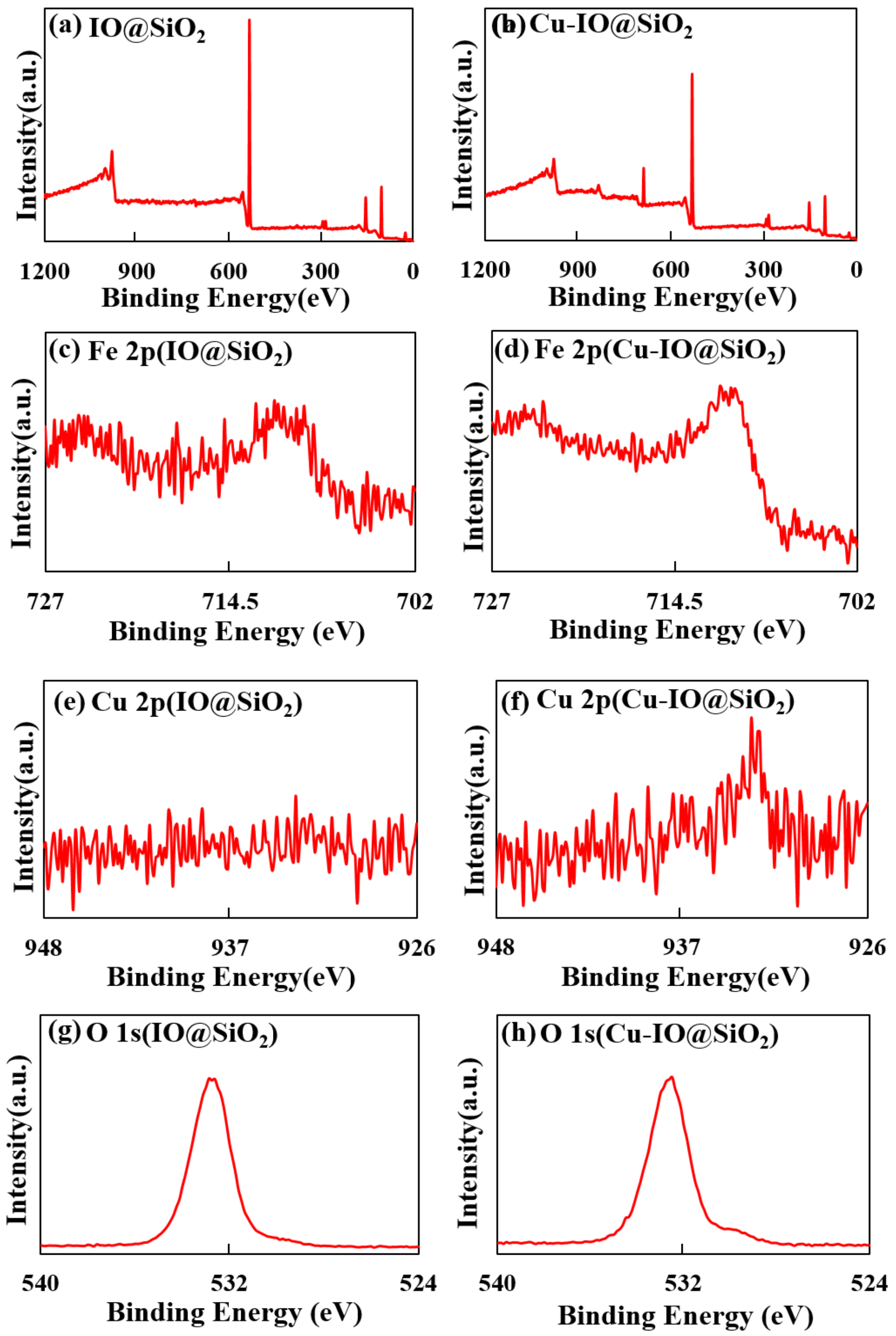

Figure S1. Survey data of (**a**) IO@SiO_2 and (**b**) Cu-IO@SiO_2. Fe 2p spectra of (**c**) IO@SiO_2 and (**d**) Cu-IO@SiO_2. Cu 2p spectra of (**e**) IO@SiO_2 and (**f**) Cu-IO@SiO_2. O 1s spectra of (**g**) IO@SiO_2 and (**h**) Cu-IO@SiO_2.

Positron Emission Tomography (PET) images of direct injection into cancer of *BALB/c* mice

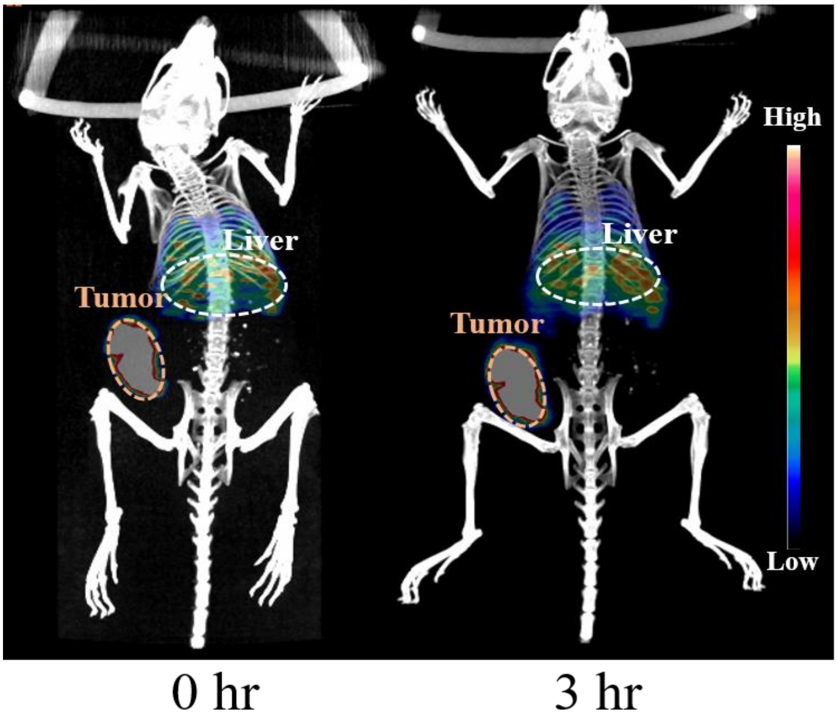

Figure S2. PET images measured immediately and in 3 h after intratumoral injection (direct injection into cancer)of the NPs for the *BALB/c* mice.
